# Identification of potential plasma biomarkers and metabolic dysfunction for unstable angina pectoris and its complication based on global metabolomics

**DOI:** 10.1042/BSR20181658

**Published:** 2019-03-22

**Authors:** Juan Wang, Wenjuan Xu, Huihui Zhao, Jianxin Chen, Bin Zhu, Xueli Li, Dong Deng, Jinping Wang, Junjie Liu, Yingting Yu, Hongbin Xiao, Wei Wang

**Affiliations:** 1 Beijing University of Chinese Medicine, Beijing 100029, China; 2 School of Life Sciences, Beijing University of Chinese Medicine, Beijing 100029, China; 3 Shanghai University of Traditional Chinese Medicine, Shanghai, 201203, China; 4 Beijing Key Laboratory of Syndrome and Prescription Basic Research, Beijing University of Chinese Medicine, Beijing 100029, China; 5 Research Center of Chinese Medicine Analysis and Transformation, Beijing University of Chinese Medicine, Beijing 100029, China

**Keywords:** diagnostic biomarker, Metabolomics, tryptophan metabolism, unstable angina pectoris, unstable angina pectoris complicated with diabetes mellitus

## Abstract

Unstable angina pectoris (UA) is one of the most dangerous clinical symptoms of acute coronary syndrome due to the risk of myocardial ischemia, which can lead to high morbidity and mortality worldwide. Though there are many advantages in understanding the pathophysiology of UA, the identification of biomarkers for the diagnosis, prognosis, and treatment of UA remains a challenge in the clinic. A global metabolomics research based on ultra-performance liquid chromatography (UPLC) combined with Q-TOF/MS was performed to discover the metabolic profile of health controls, UA patients, and UA patients with diabetes mellitus (DM), and screen for potential biomarkers. Twenty-seven potential biomarkers were determined using pattern recognition. These biomarkers, which include free fatty acids, amino acids, lysoPE and lysoPC species, and organic acids, can benefit the clinical diagnosis of UA. Pathway analysis indicated that arginine and proline metabolism, glycerophospholipid metabolism, and purine metabolism were affected in the UA patients, uniquely. Additionally, alterations in the metabolic signatures between UA and UA-complicated DM were also explored. As a result, six differential metabolites with an area under the curve (AUC) of more than 0.85 were identified as biomarkers for the diagnosis of UA and UA complicated with DM. Pathway analysis implied tryptophan metabolism was a key metabolic pathway in UA patients with DM, which provides new insights into the pathological study and drug discovery of UA.

## Introduction

Unstable angina pectoris (UA), is a clinical manifestation of acute coronary syndrome (ACS), which presents as ST-segment elevation myocardial infarction (STEMI) or non-STEMI (NSTEMI). Resulting from platelet adhesion due to the rupture of fibrous plaques on the surface of the atheroma, UA occurs secondary to acute exacerbation of coronary occlusion. Compared with stable angina, UA is more painful with longer episodes, occurs spontaneously at rest (onset angina) and more frequently, which is also a progressively worsening disorder [1,2]. However, the current diagnosis of UA depends on symptoms interpreted by patients. These symptoms include dyspnea, vomiting, sweating, fatigue, dizziness or sudden weakness, and pain or pressure in the chest, neck, jaw, abdomen, back, shoulders, or arms. Unlike other angina patterns, the above symptoms, which usually appear at rest, suddenly become more frequent or prolonged in UA patients, and do not change with rest or nitroglycerin. Besides, the release of myocardial necrosis enzymes during the progress is hardly detected [3].In addition, the patient’s case history and diagnosis are generally considered to be more sensitive and specific to UA than physical examination for diagnosis, and resting 12-lead electrocardiography (ECG) has been applied to evaluate the patient’s vital signs and symptoms and perform a cardiac classification for several years. Therefore, more effective and objective diagnosis methods are desperately needed for UA patients on a global scale. Despite more than a century of extensive research, the molecular pathophysiological process highlighting this complex ACS remains unclear.

Metabolomics has its advantages in high throughput metabolite analysis and is feasible to apply in disease diagnosis and mechanism research. Integrating bioinformatics and biostatistics, metabolomics enables the quantification and identification of biological molecules to explore the response in an organism to a disease [4]. Previous studies have demonstrated that decreasing endogenous antioxidant defenses and increasing production of reactive oxygen species are common features in UA patients [5]. Recent studies indicated that UA is a global metabolic disturbance rather than a disturbance of individual biomarkers, which suggests the need for an integrated understanding of the role of metabolites in its pathophysiology [6]. Therefore, metabolomics is the most effective approach to describe the metabolic patterns, which have the potential ability to identify patients at risk of developing unstable coronary artery disease (CAD).

Diabetes mellitus (DM), which can aggravate the deterioration of cardiac function, is one of the main complications of UA. Distinguishing the features of UA from the features of UA complicated with DM will have a benefit on the precision treatment of UA and slowdown the progression of UA. However, little attention has been given to the identification of biomarkers and pathological metabolisms of UA complicated with DM. Therefore, in the present study, the biomarkers of UA and UA complicated with DM were identified based on global metabolomics. Furthermore, the metabolic differences between UA and UA complicated with DM were also investigated systematically. The results of the present study will assist in the diagnosis of UA and UA complicated with DM in the clinic, and provide further understanding for the pathophysiologic process of UA from the initial occurrence through progression.

## Materials and methods

### Demographic baseline features

The protocol of the present study was approved by The Ethical Committee of Beijing University of Chinese Medicine (No. 81302914), and informed consent of the protocol was obtained from all participants. Patients (n = 40) were recruited from the Beijing Anzhen Hospital and were diagnosed with UA between January 2016 and September 2016. The diagnostic criteria of UA included: (1) severe and newly developed angina; (2) angina pectoris occurs at rest or with minimal exertion; and/or (3) angina with crescendo pattern. Healthy controls (n = 39) were recruited from the medical center of an affiliated hospital of the Beijing University of Chinese Medicine. The exclusion criteria included liver disease, vascular disease, cancer, thyroid disease, renal disease, and acute/chronic inflammatory disease. The patient and control participants in the present study were well-matched, and the detailed information is shown in Table 1.

**Table 1:** Basic information of clinical patient samples

	Cases UA (n = 39)	SD	Range	Controls (n = 40)	SD	Range	*P*-value
Age	43	7	25–70	45	4	23–65	0.12
Sex	19:20			18:22			0.74
Canadian Cardiovascular Society							
Class	2			N/A			
Coronary artery stenosis: non-stenosis	29:10			N/A			
BMI(mean)	25	3	17–33	21	2	18–25	0.54
Smoker: non-smoker	18:21			N/A			
**Complication**							
Hyperlipidemia: non-hyperlipidemia	21:19			N/A			
Hypertensive: non-hypertensive	6:33			N/A			
DM: non-DM	5:34			N/A			
**Physical Chemistry Index**							
Na^+^ (mean)	139	4	126–145	141	2	139–146	0.05
K^+^ (mean)	4	1	1.5–4.5	4	0	2.1–4.3	0.05
Urea (mean)	7	2	3–14	6	1	4–8	0.13
Creatinine (mean)	101	32	43–229	67	13	52–95	0.01
Hemoglobin (mean)	125	21	76–165	120	29	68–148	0.38
C-reactive protein (mean)	6.17	2	4.33–10.56	N/A			
**Drug information**							
β-blockers Y:N	14:25			N/A			
ACE inhibitors Y:N	12:27			N/A			
Nitroglycerin Y:N	38:1			N/A			
Aspirin Y:N	33:6			N/A			
Clopidogrel Y:N	23:16			N/A			
Herb Medcine Y:N	38:1			N/A			
CCB Y:N	10:29			N/A			

### Materials

HPLC-grade methanol and acetonitrile were purchased from Burdick & Jackson (USA). Formic acid (98%) was purchased from Sigma-Aldrich (USA). Ultrapure water was prepared from the Milli-Q system (Millipore, USA).

### Plasma collection and preparation

The blood samples from the participants were collected on the same day of inclusion. Patient information, including gender, age, electrolyte, ejection fraction, creatinine, BNP, hemoglobin, urea, fasting blood glucose, platelet count, high-sensitivity C-reactive protein (hs-CRP), triglyceride, total cholesterol, and medication, was recorded at inclusion. Associated diabetes, dyslipidemia, or hypertension were also added to the patient record. The venous blood of the participants was collected with EDTA as an anticoagulation and centrifuged at 3500 rpm for 15 min. Plasma samples were divided into equal aliquots and stored at −80°C. The plasma samples went through no more than two freeze-thaw cycles before metabolomics analysis.

The metabolites extraction was performed following a previous protocol with some modifications [7–9]. An aliquot of 150 μl from each plasma sample was extracted with 750 μl of precooling methanol and vortexed thoroughly for 3 min. Subsequently, let the mixture stand for 20 min. After centrifuging at 14,000 rpm for 20 min at 4°C, the upper layer (500 μl) was collected, dried by nitrogen, and then reconstituted with 150 μl of 5% acetonitrile. After centrifuging at 14000 rpm for 20 min, the supernatant was collected for LC–MS analysis.

### Plasma metabolite analysis

Plasma metabolites were acquired using Agilent 1290 ultra-performance liquid chromatography (UPLC) equipped with 6540 Q-TOF/MS. Zorbax Eclipse plus C18 chromatography column (1.8 μm, 3.0 × 100 mm; Agilent) was adapted for chromatography separation. Acetonitrile (solvent B) and water (solvent A) with 0.1% formic acid were used as the mobile phase with a gradient program as follows: 0–2 min, 5% B; 2–4 min, 5% B to 40% B; 4–5 min, 40 to 50% B; 5–10 min, 50% B; 10–15 min, 50 to 70% B; 15–19 min, 70 to 80% B. The flow rate was 0.8 ml/min, and the injection volume was 5 μl. The column temperature was 40°C. MS was performed in ESI and positive ion mode. The MS parameters were as follows: the mass range was from 100 to 1700; voltage was 3.5 kV; dry gas flow rate was 5 l/min; nebulizer gas was set at 40 psig; capillary temperature was 350°C; internal reference was used for the real-time correction to ensure the stability and accuracy of instrument. Quality control (QC) samples were obtained by pooling 20 µl aliquots of all plasma samples for assessing the quality of the metabolomics workflow. All of the samples were analyzed randomly.

### Data processing

XCMS online [10] (https://xcmsonline.scripps.edu) was applied for data preprocessing. First, the raw data from the MS were converted to mzData files and then analyzed using XCMS. The important XCMS parameters are shown below: polarity, positive mode; retention time format, 60 min; ppm, 30; minimum peak width and maximum peak width, 10 and 60, respectively; signal/noise threshold, 6; mzdiff, 0.01; prefilter intensity, 500; and profStep, 0.5. Metabolomics data were preprocessed through peak discrimination, filtering, and alignment, and normalized using probabilistic quotient normalization (PQN), log transformation, and Pareto scaling based on MetaboAnalyst 3.0 [11] (http://www.metaboanalyst.ca/MetaboAnalyst/). Pattern recognition was performed using orthogonal partial least squares discriminant analysis (OPLS-DA) with SIMCA-P 13.0 software (Umetrics, Sweden). Significance analysis was performed on SPSS Statistics17.0 software. Heatmap of specific differential metabolites was visualized using MeV software, and the classification performance (specificity and sensitivity with the highest accuracy) was assessed by the receiver operating characteristic (ROC) curve based on the SPSS 17.0 statistics software.

### Metabolic pathway and network analysis

Additionally, MetaboAnalyst 3.0 was used for metabolic pathway analysis. Other open databases, including Kyoto Encyclopedia of Genes and Genomes (KEGG) and Human Metabolome Database (HMDB), were applied for related metabolic pathways analysis. Metscape, a plug-in for Cytoscape [12], was applied to visualize and elucidate the metabolic network of specific differential metabolites.

## Results

### Global pattern analysis of plasma metabolites

**Figure 1:**
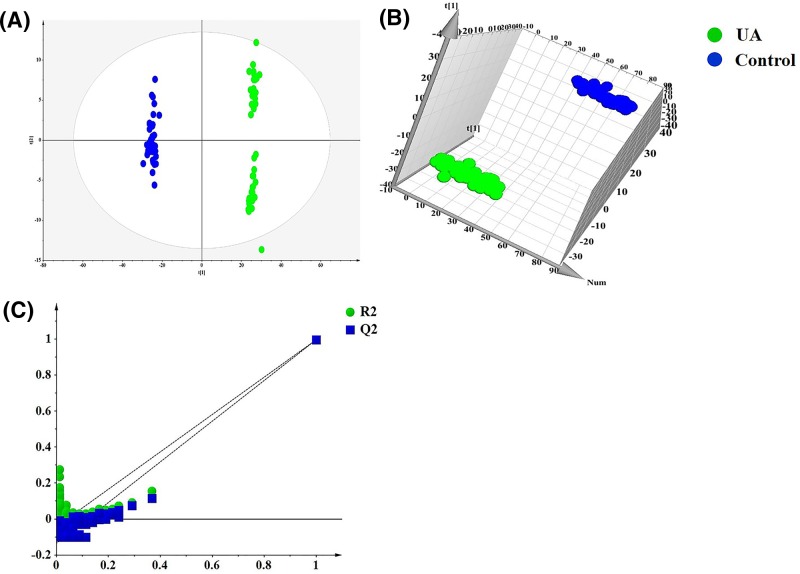
Pattern recognition results of patients with UA and control subjects (**A**) PCA-DA result, *R^2^X* = 0.72, *Q^2^* = 0.713; (**B**) OPLS-DA scatter plot, *R^2^X* = 0.72, *R^2^Y* = 0.997, *Q^2^* = 0.977; (**C**) Permutation validation plots.

The metabolic disturbances in UA patients were analyzed using a global metabolomics strategy. Obvious chromatographic differences were observed in the control, UA, and UA with complicated DM. The QC results, which indicated that the method was reliable, are shown in Supplementary Figure S1. Variables with coefficient of variation percent (CV%) more than 30% in each group were removed from further statistical analysis. For detecting subtle metabolomics differences between UA patients and the healthy controls, PCA-DA and OPLS-DA were performed as pattern recognition methods to discriminate between the above two groups (Figure 1A,B). The OPLS-DA score plot shows that there were great discrepancies between the UA patients and healthy controls (*R^2^X* = 0.72, *R^2^Y* = 0.997, *Q^2^* = 0.977), which verifies the validity of the original mode. A response permutation test with 200 iterations was adopted to avert over fitting of the OPLS-DA model (Figure 1C).

### Potential biomarkers identification of UA patients and healthy controls

**Figure 2:**
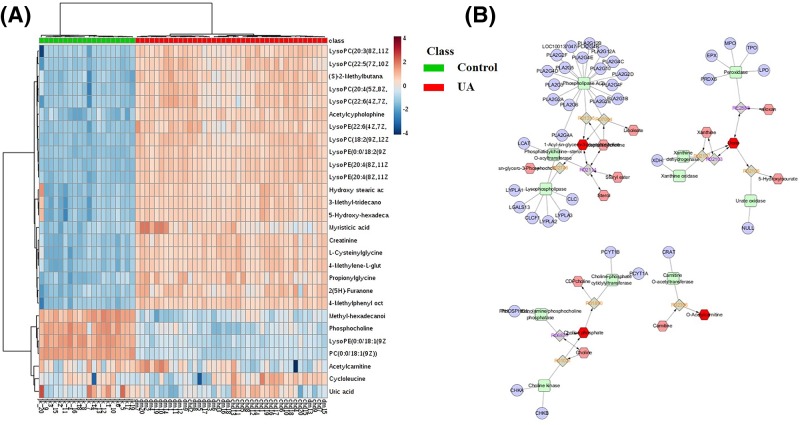
Biomarker identification and pathway analysis (**A**) Heat map of potential biomarkers obtained based on peak intensity; (**B**) Disease network construction.

Screening for candidate biomarkers remains a great challenge in improving UA clinic diagnosis and evaluating the therapeutic effect in future. In the present study, an OPLS-DA model was employed to explore the intrinsic differences between the UA patients and healthy controls and identify the potential biomarkers. As presented in Figure 1B, the OPLS-DA scatter plot shows a division between UA patients and healthy controls with a clear separation. Twenty-seven metabolites, including mostly of amino acids, lysophosphatidylcholine (lysoPC) and lysophosphatidylethanolamine (lysoPE) species, organic acids, and free fatty acids (FFAs), were detected as potential biomarkers for assisting the clinic diagnosis of UA, according to their variable importance of projection (VIP) values (VIP>1) and the Mann–Whitney *U* test (*P*< 0.05) (Table 2). Heat maps in Figure 2A illustrate the discriminative ability of each biomarker. UA is a complex holistic disorder involving many metabolites and biochemical pathways; thus, multiple biomarkers could be more powerful for diagnosis and exploring the metabolic mechanism of UA.

**Table 2:** Candidate biomarkers of patients with UA and control subjects screened by OPLS-DA (VIP value > 1.0)

Identification	HMDB IDs	m/z	Formula	Δppm	*P*-value	Fold change	VIP values
Methyl-hexadecanoic acid	HMDB61859	271.2631	C_17_H_34_O_2_	0	1.12E-27	0.41	1.9363
Cystinylglycine	HMDB00709	315.0799	C_8_H_15_N_3_O_5_S_2_	2	1.94E-30	1.80	1.8903
Propionylglycine	HMDB00783	132.0654	C_5_H_9_NO_3_	0	4.26E-08	1.22	1.81453
2(5H)-Furanone	HMDB32330	102.0548	C_4_H_7_NO_2_	1	1.45E-13	1.80	1.88709
4-Methylphenyl octanoate	HMDB37710	235.1691	C_15_H_22_O_2_	0	3.91E-33	2.88	1.65923
Creatinine	HMDB00562	114.0661	C_4_H_7_N_3_O	0	2.40E-26	2.39	1.66512
4-Methylene-L-glutamate	HMDB29433	160.0603	C_6_H_9_NO_4_	0	1.08E-30	2.27	1.54896
12-Methyl-tridecanoic acid	HMDB31072	246.2429	C_14_H_28_O_2_	0	2.23E-21	2.54	1.53262
5-Hydroxy-hexadecanoic acid	HMDB0112184	290.2689	C_16_H_32_O_3_	0	7.54E-19	2.23	1.73917
Hydroxystearic acid	HMDB62549	318.3003	C_18_H_36_O_3_	0	7.95E-14	1.91	1.91949
Acetylcypholophine^1^	C10564	378.2406	C_20_H_28_N_2_O_4_	4	2.89E-23	6.66	1.54375
LysoPC(18:2)	HMDB10386	542.3215	C_26_H_50_NO_7_P	0	1.44E-16	14.9	1.58752
Phosphocholine	HMDB01565	184.0733	C_5_H_14_NO_4_P	0	1.46E-21	0.28	1.56932
LysoPE(20:4)	HMDB11518	502.2928	C_25_H_44_NO_7_P	0	4.90E-20	7.36	1.56313
LysoPE(22:6)	HMDB11526	526.2928	C_27_H_44_NO_7_P	0	1.23E-12	4.35	1.59866
LysoPC(20:4)	HMDB10395	544.3399	C_28_H_50_NO_7_P	0	1.98E-15	7.36	1.58752
LysoPE(0:0/18:2)	HMDB11477	478.2923	C_23_H_44_NO_7_P	1	1.01E-14	78.9	1.72129
(S)-2-Methylbutanal	HMDB31525	104.1069	C_5_H_10_O	0	2.11E-16	2.97	1.90524
LysoPC(22:6)	HMDB10404	568.3399	C_30_H_50_NO_7_P	0	5.36E-12	4.35	1.90152
LysoPC(20:3)	HMDB10394	546.3552	C_28_H_52_NO_7_P	0	1.57E-13	10.7	1.74299
LysoPC(22:5)	HMDB10403	570.3555	C_30_H_52_NO_7_P	0	2.95E-13	9.55	1.52973
LysoPE(0:0/18:1)	HMDB11476	480.3083	C_23_H_46_NO_7_P	0	5.59E-22	0.07	1.5225
PC(0:0/18:1)	HMDB62651	522.3547	C_26_H_52_NO_7_P	1	1.79E-30	0.07	1.62132
Acetylcarnitine	HMDB00201	204.0599	C9H_17_NO_4_	0	2.32E-06	0.73	1.87673
Myristicic acid	HMDB30800	219.0243	C_9_H_8_O_5_	8	1.15E-07	1.47	1.51825
Uric acid	HMDB00289	169.0337	C_5_H_4_N_4_O_3_	0	1.02E-07	0.67	1.5791
Cycloleucine	HMDB62225	130.0861	C_6_H_11_NO_2_	1	0.02068	0.96	2.1655

1 KEGG ID, for the metabolites that have no HMDB ID.

### Pathway analysis and network construction

Metabolic pathway analysis has practical significance for systematically evaluating the metabolic status of disease and revealing the related metabolic functions. This analysis indicated that three pathways were found to be obviously affected in UA patients, including glycerophospholipid metabolism, arginine and proline metabolism, and purine metabolism. The detailed results of this pathway analysis are shown in Table 3. A disease metabolic network can display the complex interaction between genes, proteins, metabolites, and drugs. In the present study, the metabolic network of UA was reconstructed according to the potential biomarkers of UA patients by Cytoscape (Figure 2B). There were 75 nodes and 78 edges in the UA-related network, and the network density and heterogeneity were 0.028 and 1.137, respectively. These results indicated that UA is a network phenomenon. Constructing UA-related metabolic networks may have a high intrinsic potential for disease diagnosis and drug discovery in the future.

**Table 3:** Results of pathway analysis

Pathway	Match status	Metabolites	Raw p	-log(p)	Holm adjust	FDR	Impact
Glycerophospholipid metabolism	2/39	Phosphocholine, LysoPC(18:2)	2.37E-44	1.00E+02	7.12E-44	7.12E-44	0.05
Arginine and proline metabolism	1/77	Uric acid	1.36E-32	7.34E+01	2.72E-32	2.04E-32	0.01
Purine metabolism	1/92	Creatinine	6.11E-02	2.80E+00	6.11E-02	6.11E-02	0.01

### Alteration in metabolic signature between UA and UA complicated DM

**Figure 3:**
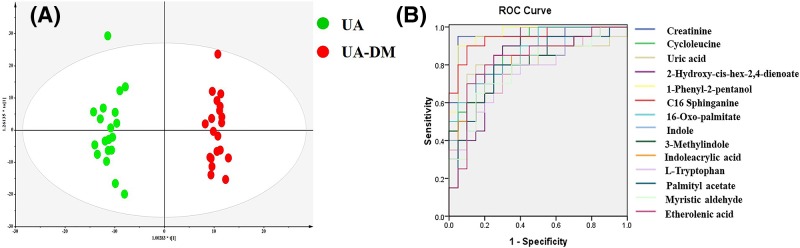
Biomarkers identification and validation of UA and UA complicated with DM **(A)** OPLS-DA scatter plot, *R^2^X* = 0.268; *R^2^Y* = 0.981; *Q^2^* = 0.817; **(B)** Evaluation of potential biomarker based on ROC analysis.

DM, which aggravates the deterioration of cardiac function, is one of the most common complications in CAD and also a risk factor for UA. UA complicated with DM is more likely to progress to a myocardial infarction; therefore, prevention and treatment are particularly important. In the present study, the differential metabolism between UA and UA complicated with DM has been explored, providing valuable references for clinical diagnosis and precise treatment. Similar to UA, the potential metabolic biomarkers between UA and UA complicated with DM were identified through OPLS-DA pattern recognition (Figure 3A). Statistical analysis determined a distinct separation between UA patients and UA complicated with DM (*R^2^X* = 0.268, *R^2^Y* = 0.981, *Q^2^* = 0.817). The characteristic biomarkers of UA complicated with DM are listed in Table 4, and the biomarkers, which may be helpful to improve the diagnosis of UA complicated with DM, are visualized in the box plots in Supplementary Figure S2. There were 22 biomarkers that were different between UA and UA complicated with DM, including eight upregulated metabolites and 14 downregulated metabolites. The box plot shows that (E)-10,11-dihydro-alpha-atlantone, 2-heptoxyethanethiol, D-fuconate, PS(O-18:0/0:0), and myristicic acid were the main upregulated biomarkers, while hexadecasphinganine and creatinine were the main downregulated ones. To our knowledge, this is the first report of biomarkers screened for UA and UA complicated with DM, which could accelerate the precision medicine process. The diagnostic performance of each metabolite was evaluated using a ROC curve. Area under the curve (AUC) values with 95% confidence interval, sensitivity, and specificity are shown in Figure 3B and Table 5. Six out of twenty-two differential metabolites achieved an AUC of more than 0.85 with high sensitivity and specificity. As a result, creatinine, cycloleucine, 1-phenyl-2-pentanol, C16 sphinganine, and 16-oxo-palmitate have the potential to be non-invasive diagnosis metabolites of UA and UA complicated with DM.

**Table 4:** Candidate biomarkers of patients with UA and UA complicated with DM

Identification	HMDB IDs	m/z	Formula	*P*-value	Fold change	VIP values
Creatinine	HMDB00562	114.066	C_4_H_7_N_3_O	2.03E-08	0.81	2.48247
Cycloleucine	HMDB62225	130.0861	C_6_H_11_NO_2_	1.35E-05	0.42	2.09198
Myristicic acid	HMDB30800	219.0245	C_9_H_8_O_5_	3.12E-04	1.28	1.89559
Acetylcarnitine	HMDB00201	204.1229	C_9_H_17_NO_4_	1.67E-03	1.29	1.61667
Uric acid	HMDB00289	169.0355	C_5_H_4_N_4_O_3_	5.56E-05	0.75	1.73531
^1^2-Hydroxy-cis-hex-2,4-dienoate	C11354	151.0366	C_6_H_8_O_3_	4.84E-06	0.83	2.04841
2-Heptoxyethanethiol	HMDB32380	185.0991	C_8_H_18_OS	3.01E-07	2.11	2.64919
(E)-10,11-Dihydro-alpha-atlantone	HMDB36201	241.1563	C_15_H_22_O	8.30E-06	1.89	2.17376
^1^Hexadecasphinganine	C13915	274.2757	C_16_H_35_NO_2_	9.33E-09	0.76	2.5634
16-Oxo-palmitate	C19614	288.2556	C_16_H_30_O_3_	8.76E-06	0.74	2.11338
^2^PS(O-18:0/0:0)	LMGP03060002	512.3375	C_24_H_50_NO_8_P	7.00E-07	2.04	2.47083
^1^D-Fuconate	C01680	203.0538	C_6_H_12_O_6_	6.57E-04	1.49	1.86809
^2^9Z,11E,13-Tetradecatrienal	LMFA06000182	229.1563	C_14_H_22_O	1.165E-03	0.82	1.52906
Indole	HMDB00738	118.0659	C_8_H_7_N	0.76E-03	0.80	1.84156
3-Methylindole	HMDB00466	132.0817	C_9_H_9_N	1.14E-03	0.82	1.81453
Indoleacrylic acid	HMDB00734	188.0715	C_11_H_9_NO_2_	1.38E-03	0.81	1.73732
L-Tryptophan	HMDB00929	205.0983	C_11_H_12_N_2_O_2_	3.45E-03	0.84	1.57986
^1^N-Acetyldemethylphosphinothricin	C17949	227.0807	C_6_H_12_NO_5_P	3.79 E-03	1.35	1.8096
^2^Palmityl acetate	LMFA07010379	285.2808	C_18_H_36_O_2_	1.74 E-03	0.76	1.74697
12-Methyl-tridecanoic acid	HMDB31072	246.2447	C_14_H_28_O_2_	6.01E-03	1.16	1.53262
Myristic aldehyde	HMDB34283	230.2497	C_14_H_28_O	4.95E-06	0.86	1.65054
^1^Etherolenic acid	C16319	310.2375	C_18_H_28_O_3_	4.44E-08	0.88	1.8903

1 KEGG ID, for the metabolites that has no HMDB ID.

2 LipidMaps ID, for the metabolites that has no HMDB ID and KEGG ID.

**Table 5:** ROC analysis results of candidate biomarkers of patients with UA and UA complicated with DM

Variable(s)	AUC	Std. error	Asymptotic 95% confidence Interval		Sensitivity	Specificity
			Lower	Upper		
1-Phenyl-2-pentanol	0.96	0.03	0.00	1.00	0.90	0.95
Creatinine	0.95	0.04	0.00	1.00	0.95	0.90
C16 Sphinganine	0.95	0.03	0.00	1.00	0.90	0.90
Cycloleucine	0.88	0.05	0.77	0.98	0.90	0.70
16-Oxo-palmitate	0.87	0.05	0.76	0.97	0.75	0.85
2-Hydroxy-cis-hex-2,4-dienoate	0.85	0.06	0.73	0.97	0.85	0.75
Etherolenic acid	0.82	0.07	0.69	0.96	0.80	0.80
Uric acid	0.81	0.07	0.67	0.96	0.75	0.90
Indole	0.81	0.07	0.67	0.94	0.70	0.85
3-Methylindole	0.81	0.07	0.67	0.94	0.80	0.75
Indoleacrylic acid	0.80	0.07	0.66	0.93	0.85	0.65
Palmityl acetate	0.80	0.07	0.65	0.94	0.80	0.75
Myristic aldehyde	0.78	0.07	0.64	0.93	0.65	0.85
L-Tryptophan	0.76	0.08	0.61	0.91	0.75	0.70

A metabolic pathway analysis was performed using the above 22 biomarkers, and three distinctive pathways were discovered for UA complicated with DM, including arginine and proline metabolism, purine metabolism, and tryptophan metabolism. Compared with the three pathways of UA, arginine and proline metabolism, purine metabolism were indicated as significant in UA and UA complicated with DM. Of the pathways, glycerophospholipid metabolism has a greater impact on UA patients and tryptophan metabolism has a greater impact on UA patients with DM. This is the first study identifying tryptophan metabolism as a critical pathway in UA complicated with DM, which will initiate further research revealing the related mechanisms.

## Discussion

Up to now, the clinical diagnosis of UA has been a challenge for the prevention and treatment of CAD. Meanwhile, advanced understanding of the metabolic alteration that occurs in CAD patients with complications is quite urgent. For example, DM is known to increase the severity of heart function. In the present study, a UPLC-QTOF/MS-based global metabolomics approach was performed on the plasma of patients with UA and UA complicated with DM to screen for potential biomarkers and reveal the pathways associated with those diseases. Additionally, the present study filled a gap in understanding UA complicated with DM and provided new insights into the biological mechanisms surrounding this disorder.

Plasma metabolomics data demonstrated that specific metabolites exhibited various patterns between UA and UA complicated with DM. Rather than evaluating the diagnostic performance of the metabolomics results, the critical metabolites discriminating patients with UA, UA complicated with DM, and healthy controls were identified. Ultimately, 27 biomarkers in UA patients and 22 biomarkers of UA complicated with DM were determined, which has great advantages in visualizing the metabolic differences between UA and UA with complications, and improving the diagnostic performance of our model. Six common biomarkers, including creatinine, 12-methyl-tridecanoic acid, acetylcarnitine, myristicic acid, uric acid, and cycloleucine, were identified between the two groups. Based on our analysis in the present study, it is reasonable to believe that the expression changes of these six metabolites have a vital impact on the onset and development of UA. Noteworthy, creatinine is phosphorylated to phosphocreatine in muscles, which is the most common indicator of renal function [13,14]. Additionally, an elevated creatinine concentration has been associated with the order of severity in CAD patients, which also indicates a physiological state that is energy-depleted [15,16]. Our data also illustrate this point. Moreover, high uric acid concentration is a risk factor for both cardiovascular disease and abnormal glucose tolerance, which is affected by various complications of metabolic syndrome [17].Meanwhile, lipid oxidation metabolism, which accounts for most of the altered metabolites in the present study, correlates with the production of oxidized low-density lipoproteins (ox-LDL) and inflammatory conditions in UA. In LDL particles, circulating lysoPC constitutes about 1–5% of the total phosphatidyl choline (PC). Moreover, during the LDL oxidation process, about 50% of PC is converted to lysoPC and FFAs by phospholipase a2 (PLA2) [18,19]. Circulating lysoPC has proatherogenic effects on monocyte recruitment, macrophage proliferation, smooth muscle cell proliferation, and endothelial dysfunction; thus, it has been regarded as one of most significant underlying pathogenic factors for atherosclerosis [20,21]. Increasing lysoPC is a metabolic feature during the oxidative changes found in CAD patients, which is consistent with previous study [20,22]. In our perspective, these potential biomarkers could guide clinical practice. The disturbances of the metabolic pathway, which was beneficial to identify the early stage of diagnosis, explore pathological mechanism, and discover new drugs.

**Figure 4:**
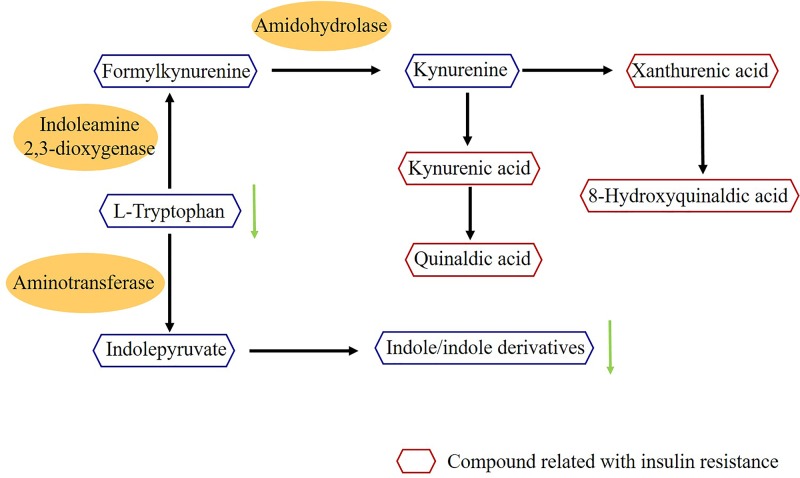
Tryptophan metabolism pathway Hexagons represent metabolites in tryptophan metabolism pathway and the red ones are compounds related to insulin resistance. The yellow ellipse represents the key.

In addition, metabolic pathways are modified by a series of enzymatic reactions, which participate in specific biological processes. Many diseases are proven to be network phenomena, and a metabolic network could help to describe the complex relationship among various kinds of metabolites, contributing to drug discovery. Here, MetPA and Metscape were used to reveal the pathways related to UA and UA complicated with DM, and construct metabolic networks separately. The pathway analysis indicated that arginine and proline metabolism, purine metabolism, and tryptophan metabolism were the distinctive pathways of UA complicated with DM. It is worth noting that tryptophan metabolism is a typical metabolic pathway of UA complicated with DM. Therefore, the key metabolites and enzymes in the tryptophan metabolism pathway were investigated systematically; the detail information is shown in Figure 4. The tryptophan metabolism pathway mainly contained two main metabolic patterns: an indole ring maintained to form indoles and tryptophan oxidation to form kynurenine. The quantitative results of the biomarkers in Table 4 showed that tryptophan and the indole metabolites (indole, 3-Methylindole, indoleacrylic acid) were down-regulated. This indicates that the metabolism of the indole pathway was decreased, while the oxidation pathway was strengthened. Notably, oxidized products of tryptophan (kynurenic acid, xanthurenic acid, quinaldic acid, 8-hydroxyquinaldic acid), labeled in Figure 4, have a direct correlation with insulin resistance [23,24]. This suggests that tryptophan metabolism could regulate insulin resistance, which also proves the biomarkers identified in the present study are reliable. Further studies on the mechanisms regarding this issue are also imperative.

## Summary

The identification of biomarkers is one of the main applications of metabolomics. In the present study, a global metabolomics strategy based on UPLC combined with Q-TOF/MS was used to screen for biomarkers of UA and UA complicated with DM. There were 27 and 22 plasma biomarkers identified in patients with UA and UA complicated with DM, respectively. The identified metabolites associated with UA were key participants in energy metabolism, lipid complexes, and amino acids, which provided a reference for clinical diagnosis. Pathway and network analysis based on the biomarkers revealed that tryptophan metabolism was a key metabolic pathway in UA patients with DM, which provided a new understanding in the biological metabolic mechanism of UA complicated with DM. It is believed that systematically exploring potential biomarkers and distinguishing the metabolic mechanism of UA from its complications will promote personalized medicine and drug discovery.

## Supporting information

**Supplementary Figure S1:** 

**Supplementary Figure S2:** 

**Table S1:** The CV-ANOVA results of OPLS-DA models.

## Abbreviations

ACS: acute coronary syndrome
AUC: area under the curve
CAD: coronary artery disease
DM: diabetes mellitus
FFA: free fatty acid
HMDB: Human Metabolome Database
KEGG: Kyoto Encyclopedia of Genes and Genomes
lysoPC: lysophosphatidylcholine
lysoPE: lysophosphatidylethanolamine
OPLS-DA: orthogonal partial least squares discriminant analysis
ox-LDL: oxidized low-density lipoproteins
PC: phosphatidyl choline
QC: quality control
ROC: receiver operating characteristic
UA: unstable angina pectoris
UPLC: ultra-performance liquid chromatography
VIP: variable importance of projection
