# Supplementary material for: Identification of potential plasma biomarkers and metabolic dysfunction for unstable angina pectoris and its complication based on global metabolomics
Source: Biosci Rep. 2019 Mar 22;39(3):BSR20181658. doi: 10.1042/BSR20181658 (PMC6430740; doi:10.1042/BSR20181658)
Supplement: Supplementary file 1 [file bsr-39-bsr20181658_Supp1.pdf]

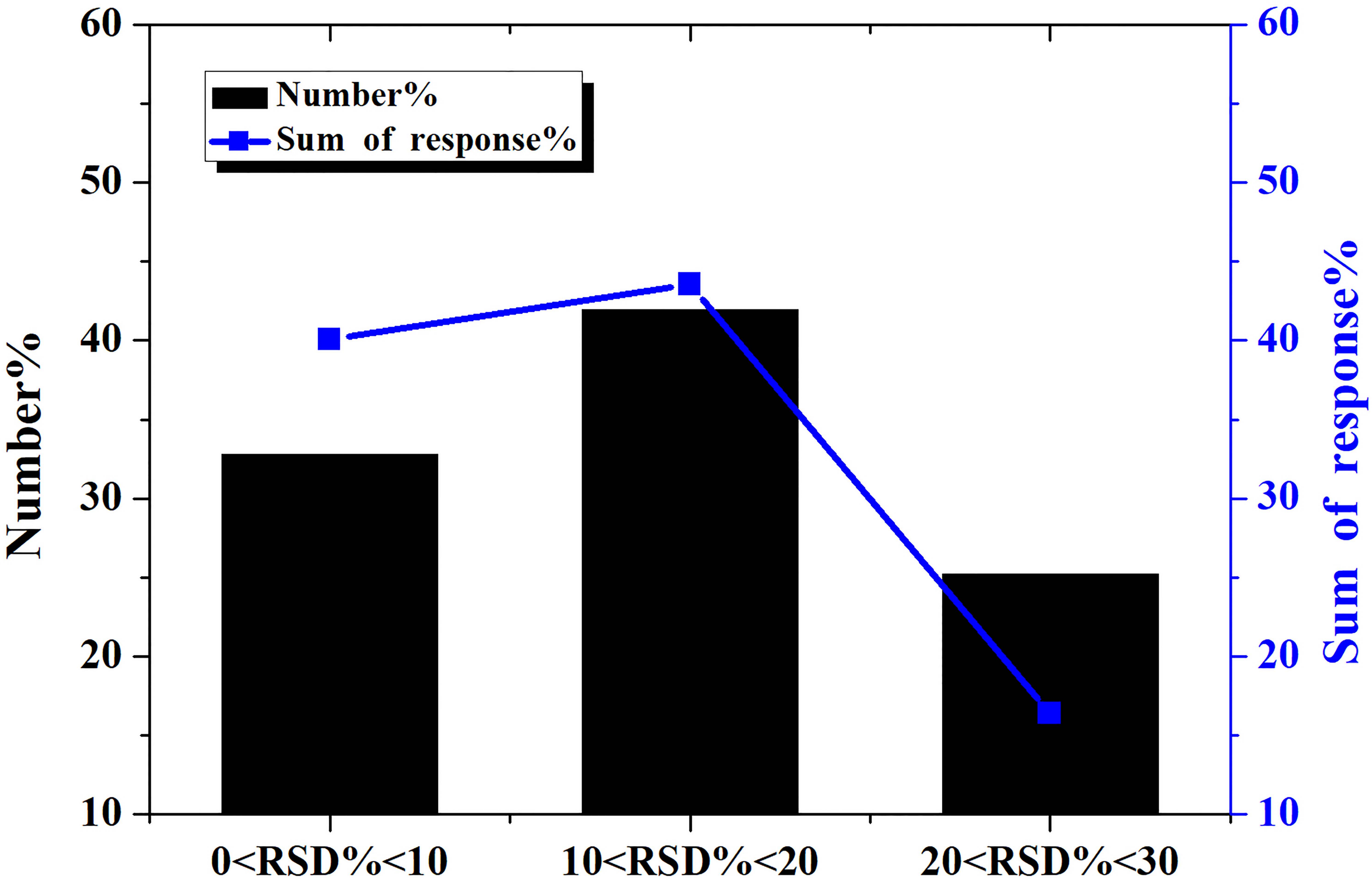

Number%
Sum of response%
60
50
40
30
20
10
0<RSD%<10
10<RSD%<20
20<RSD%<30

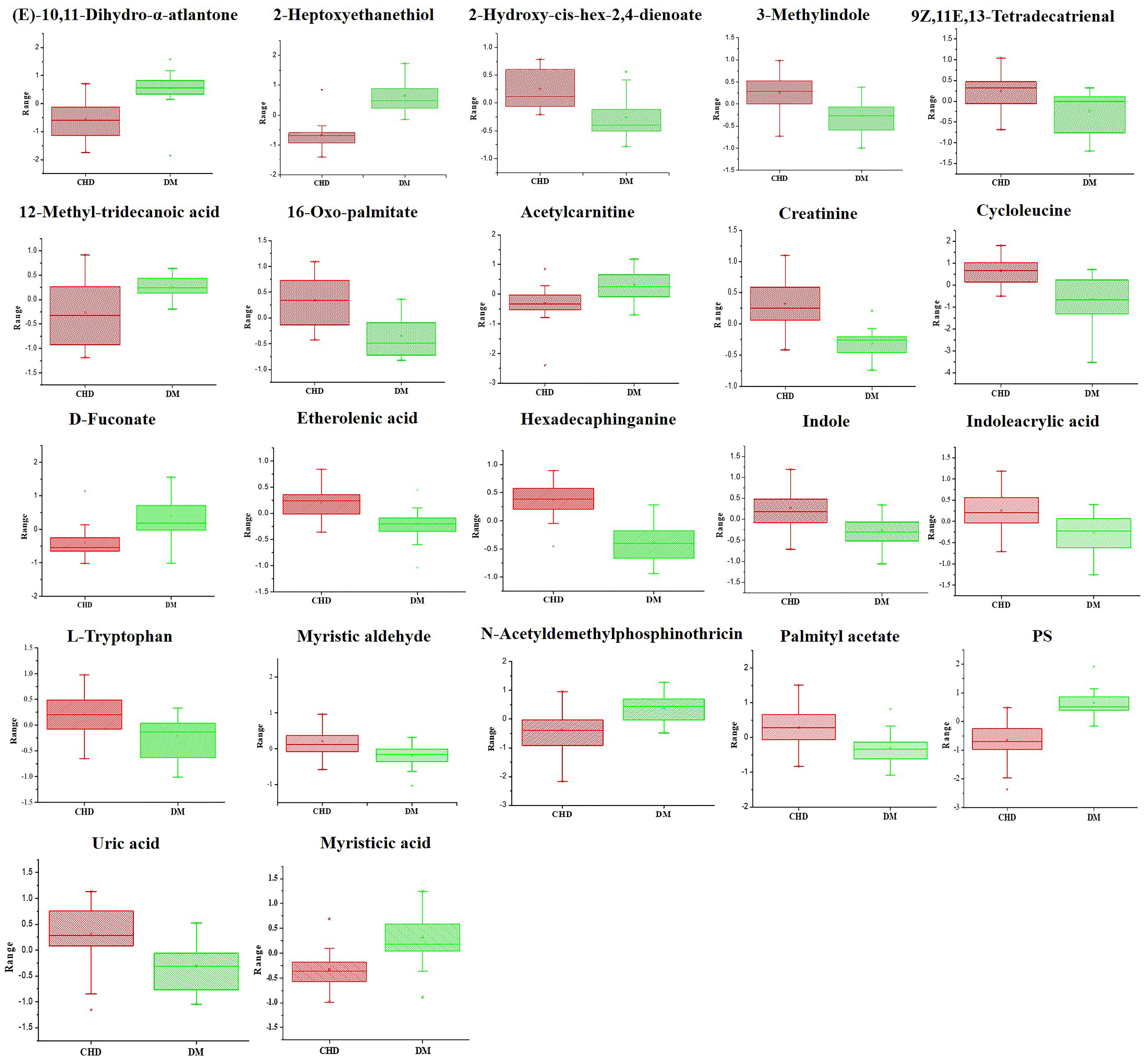

(E)-10,11-Dihydro-α-atlantone
2-Heptoxyethanethiol
2-Hydroxy-cis-hex-2,4-dienoate
3-Methylindole
9Z,11E,13-Tetradecatrienal
12-Methyl-tridecanoic acid
16-Oxo-palmitate
Acetylcarnitine
Creatinine
Cycloleucine
D-Fuconate
Etherolenic acid
Hexadecaphinganine
Indole
Indoleacrylic acid
L-Tryptophan
Myristic aldehyde
N-Acetyldemethylphosphinothricin
Palmityl acetate
PS
Uric acid
Myristicic acid
Range
CHD
DM

Table S1 The CV-ANOVA results of OPLS-DA models.

M3(Untitled)	SS	DF	MS	F	p	SD
**CHD *vs* Control**						
Total corr.	78	78	1			1
Regression	77.7406	2	38.8703	11388	0	6.2346
Residual	0.259408	76	0.00341326			0.0584232
**CHD *vs* DM**						
Total corr.	38	38	1			1
Regression	31.0626	6	5.1771	23.8802	1.59823e-010	2.27532
Residual	6.93743	32	0.216795			0.465612
